# Robust Reproducible Resting State Networks in the Awake Rodent Brain

**DOI:** 10.1371/journal.pone.0025701

**Published:** 2011-10-18

**Authors:** Lino Becerra, Gautam Pendse, Pei-Ching Chang, James Bishop, David Borsook

**Affiliations:** Pain and Analgesia Imaging Neuroscience Group, A. A. Martinos Center for Biomedical Imaging, Massachusetts General Hospital, Boston, Massachusetts, United States of America; Beijing Normal University, Beijing, China

## Abstract

Resting state networks (RSNs) have been studied extensively with functional MRI in humans in health and disease to reflect brain function in the un-stimulated state as well as reveal how the brain is altered with disease. Rodent models of disease have been used comprehensively to understand the biology of the disease as well as in the development of new therapies. RSN reported studies in rodents, however, are few, and most studies are performed with anesthetized rodents that might alter networks and differ from their non-anesthetized state. Acquiring RSN data in the awake rodent avoids the issues of anesthesia effects on brain function. Using high field fMRI we determined RSNs in awake rats using an independent component analysis (ICA) approach, however, ICA analysis can produce a large number of components, some with biological relevance (networks). We further have applied a novel method to determine networks that are robust and reproducible among all the components found with ICA. This analysis indicates that 7 networks are robust and reproducible in the rat and their putative role is discussed.

## Introduction

Resting state networks (RSNs) in humans is becoming a tool to investigate brain basal states in health and disease [1]–[7]. Using fMRI, neuronal baseline activity of the brain is thought to measure low frequency fluctuations that are related to functionally relevant networks [8]–[10]. In humans these network patterns seem to be consistent and reproducible across subjects and over time [8], [11]–[14]. Using this approach it is possible to characterize the brain networks in terms of functionally relevant circuits that subserve specific actions such as environmental monitoring, action-execution, and sensory-perception domains [11], [12], [15].

Until recently, RSNs have not been studied in rodents. Consequently, healthy state basal networks are not well understood. Most rodent studies have been carried out on anesthetized animals. Furthermore, these studies use a seed-based approach in which specific questions are tested by using a particular brain region as a seed and determining what else in the brain displays similar temporal pattern. Kannurpatti et al. [16] used the primary somatosensory cortex (S1) as seed and found a network that consisted of bilateral S1. Zhao et al. [17] (using medetomidine anesthesia) and Majeed et al. [18] (alpha-chloralose) found S1 as well as bilateral Caudate Putamen networks (CPu) connectivity. Pawela et al., [19], [20] found an extended cortical network including primary/secondary sensorimotor regions under medetomidine anesthesia. Two studies (Lu et al. [21] with alpha-chloralose and Wang et al. [22] with isoflorane) explored the effects of levels of anesthesia on networks and determined that connectivity decreased with increased levels of anesthesia. Similarly, in anesthetized monkeys, the networks observed in humans are also preserved [23]. These results have raised issues relating to the significance of the networks to reflect meaningful biology [24] since consciousness should be impaired under anesthesia. In a recent study by Liu et al. [25], in which fMRI (BOLD and CBF) as well as electrophysiology was used, they reported that while the level of anesthesia was light, good correlation between electrical activity and fMRI signals was observed. At deep levels of anesthesia, most burst electrical activity was suppressed but brain networks were still observed, although weaker than with light anesthesia. These findings were interpreted to reflect that at deep levels of anesthesia a loss of consciousness occurs due to a collapse of cortical activity patterns as reflected in the disappearance of electrical burst activity. The underlying coherent fluctuations that remain at deep levels of anesthesia are the ones that appear in fMRI studies, although they might not reflect brain activity. Hence, it is probable that varying types and levels of anesthetics affect spontaneous brain activity and the resulting RSNs.

To the best of our knowledge, there is only one other study that explores RSNs in awake rodents [26] with a seed-based correlation method. The study used 3 seeds located at the prefrontal cortex, thalamus and retrosplenial cortex. There was a significant amount of overlap in the observed networks for the 3 seeds. The cortical ribbon encompassing sensorimotor regions was in all of them, suggesting that seed regions might contain temporal information of two or more networks, perhaps due to the size of the region or because of intrinsic connectivity in many networks.

An alternative approach in determining RSNs is the application of independent component analysis (ICA - [11]) commonly used in humans. ICA does not need a-priori specifications of seed regions. It determines the optimal components that can explain the spatio-temporal patterns in the data. The obtained components might provide information that is different to the seed-based approach. For instance, a seed could be based on a brain region belonging to two or more networks. In this case, the time course extracted from this seed will be a mixture of two time courses - one from each network. A seed-based analysis of such a region might provide a resulting network that is a combination of other networks. For instance, in awake rats a seed- based connectivity analysis [26] with somatosensory cortex as a seed produced a network consisting of the sensorimotor cortex. An ICA study of awake rodents [27] had that particular network split into 2 (as we have found in this article) indicating that a structure could have activity belonging to different networks. Two studies have been reported utilizing ICA's: one in anesthetized rodents [28] and the other in awake rats [27]. Hutchison used low levels of isoflurane (1%) and determined 20 components of which they selected, visually, 12 networks for the brain structures they encompassed. Some of the networks observed by Hutchison (S1 network, S2 network, visual, auditory) appear as a single one in Zhang's awake connectivity analysis, indicating that perhaps they are separate networks. The ICA study by Liang et al. [27] also applied graph theory with the aim of understanding the connectivity of the observed networks. They determined 40 ICA components and from them selected 8 that included cortical and subcortical structures (however, the cortical ribbon network observed by their seed analysis in their previous paper seems to be split into different components). Through a graph-based analysis, they derived 3 modules that seem to correspond to sensorimotor, integration/cognitive processing, and emotion/autonomic regulation. Thus, while the field has clearly advanced the issue of RSN selection would be improved if it were determined by a more robust or objective approach.

Optimally, determination of the basic RSNs should be carried out on awake rodents to eliminate the confounding effects of anesthesia. Additionally, a model-free analysis approach such as ICA should be used to define networks. However, as mentioned above, the selection process of significant components in an ICA analysis is rather arbitrary.

We have recently extended a technique (RAICAR - [29]) to be able to statistically determine the most robust and reproducible components of an ICA-based analysis [29]).

In this article, we have acquired baseline fMRI data in conscious rats and utilized ICA to determine robust reproducible RSNs. Our results indicate that 7 components meet the criteria and their putative role in brain function is discussed.

## Methods

### Ethics Statement

All methods and experiments were approved by the Massachusetts General Hospital Subcommittee on Research Animal Care (IACUC-Protocol #2008N00192). Animals were caged 2 per cage and on a 12/12 hours light/dark cycle. Food and water were available ad libitum. Animals were inspected daily for welfare or signs of disease. No animal displayed excessive signs of stress (vocalizing trashing around) during training or imaging session.

### Rat imaging

Imaging methods have been described elsewhere [30]: rats were adapted to the imaging set-up by consecutive restraining over 3 days, one hour each day in a holding device similar to the one used for imaging set in a box with speakers playing the scanner noises, they were lightly anesthetized with 2% isoflorane for loading. On the imaging day, rats were slightly anesthetized with 2% isoflorane for loading into the imaging cradle; they were left to recover from isoflorane for 30 minutes before imaging to avoid any remnant effects of anesthesia. Fifteen Sprague-Dawley rats (males, weight, 300–350 g) were scanned in a 4.7T scanner (Bruker, Billerica, MA) in total darkness. Gradient Echo EPI images consisted of 12 slices 1.5 mm thick with a field of view of 30 mm and in-plane resolution of 64×64 voxels covering from the front of the brain up to most of the cerebellum. Images were acquired with TE/TR = 12 ms/3 s, 90° flip angle and 120 time points (6 minutes) per rat. We used a shorter TE to reduce susceptibility-induced distortions around the ear canals. However, a short TE reduces the BOLD contrast and might impair reproducibility of some networks. Two rats displayed excessive head-motion (>0.5 mm) and were excluded from further analysis.

### Analysis

Analysis was performed using tools from the FMRIB software library (http://www.fmrib.ox.ac.uk/fsl/). The following pre-processing steps were carried out: (i) *Motion correction and spike detection*: Each rat was subjected to motion correction using FSL's motion correction algorithm MCFLIRT and spike detection using FSL's *fsl_motion_outliers* script. (ii) *Brain extraction:* After motion correction, for each rat masks were drawn to delineate the brain tissue from the background. Brain extraction was done manually because FSL's brain extraction tool BET was not able to automatically remove the non-brain tissue in this case. (iii) *Data cleanup:* Motion and spike related artifacts were removed from the data by regressing out simultaneously the 6 motion parameters and the spike explanatory variable (EVs) created in step (i). The spike EVs are all 0 except at the volume location where a spike is detected. (iv) *Spatial smoothing and band pass filtering:* The cleaned data from step (iii) was preprocessed by applying a spatial smoothing using a Gaussian kernel FWHM of 0.9 mm, a grand mean intensity normalization of the entire data set using a single multiplicative factor, and band pass temporal filtering using filter cutoffs of 100 and 10 s (0.01–0.1 Hz) (similar to Liang et al. 2011). (v) *Normalization to standard space:* Registration of fMRI space data to standard space was carried out using FSL's flirt using a 12 degrees of freedom affine transform. The standard space was chosen to be an anatomical scan from 1 rat of size 256×128×24 with voxel size 0.117×0.117×1 mm^3^. To reduce computational burden in the resting state analysis the 4-D filtered datasets from step (iv) were resampled to spatial size 100×75×48 with voxel size 0.3×0.2×0.5 mm^3^, a similar approach used for human analysis [11].

### Resting State Analysis

Resting state analysis was performed using spatial independent component analysis (ICA) using the tool MELODIC from FSL. There are 2 sources of variability when doing ICA. First, ICA results are produced by optimization of a non-convex function, which means that the results are not unique. Indeed, running ICA several times on the same dataset gives different results. Second, there is inter-subject variability in the ICA results. Given these sources of variability, we would like to find ICA components that are good descriptors of the resting state as well being reproducible across runs and across subjects.

Given our initial population of 13 rats (2 out of 15 were excluded due to excessive motion), we randomly picked 5 rats without replacement out of 13 to create a group of 5 rats. We repeated this process to create 50 different groups of 5 rats. Each group of 5 rats was subjected to a temporal con-catenation based group ICA (concat-ICA). The number of independent components in concat-ICA was fixed at 20 (similar to human RSN studies [12]). One advantage of group concat-ICA analysis is that we constrain the group to have the same spatial maps but allow flexibility in modeling subject specific associated time-courses.

For reproducibility of RSN analysis we adopted the following approach. The 50 group ICA maps from individual 5 subject concat-ICAs were subjected to a ranking and reproducibility analysis (RAICAR, [29]). The reproducibility analysis is a very efficient technique for filtering out only those ICA components that are reproducible across the input data sets. In our case, since the 50 groups of 5 are chosen randomly from 13 rats, the reproducibility is over multiple runs and subjects simultaneously.

In the original RAICAR algorithm, there is no well-defined cutoff on reproducibility. Hence, we modified the RAICAR algorithm to have an automatically determined reproducibility cutoff [31]. Briefly, the cross-realization correlation matrix (CRCM) in RAICAR was randomly permuted 100 times and the RAICAR analysis was repeated for each random permutation. This generated a null-distribution for the reproducibility index, which was used to compute reproducibility p-values for each independent component.

For group averaging and thresholding, the reproducible components (e.g., p-value<0.05) across the 50 groups ICA runs were automatically preserved as described above. These components were then analyzed voxel-wise using a group mean general linear model (GLM). A mixture model (MM) was fitted to the resulting t-statistic maps using algorithms developed in MATLAB [32] to determine corrected thresholds. The “activated” and “deactivated” components of the MM were modeled by a Gamma distribution and a “flipped” Gamma distribution, respectively. The null distribution was modeled using a Student t distribution. Non-background components were detected by thresholding the posterior probability maps for “activation” and “deactivation” at posterior probability >0.5. Note that because of “sign ambiguity” in ICA “positive” or “negative” activation simply indicates reversed polarity of modulation.

### Component (Network) Identification

Each component was analyzed to determine brain structures, or parts thereof, that were covered by the network. In addition to the component average statistical value for each brain structure, the percentage of the volume of the structure in the network was calculated. These two metrics were used to classify brain structures within a component in order of statistical significance and relative fraction of the structure involved; components were assigned a specific biological function based on the most salient brain structures in the network.

Brain structures were identified on MRI slices that correspond to a standard Atlas [33], [34].

## Results

### Methodological

**Figure 1:**
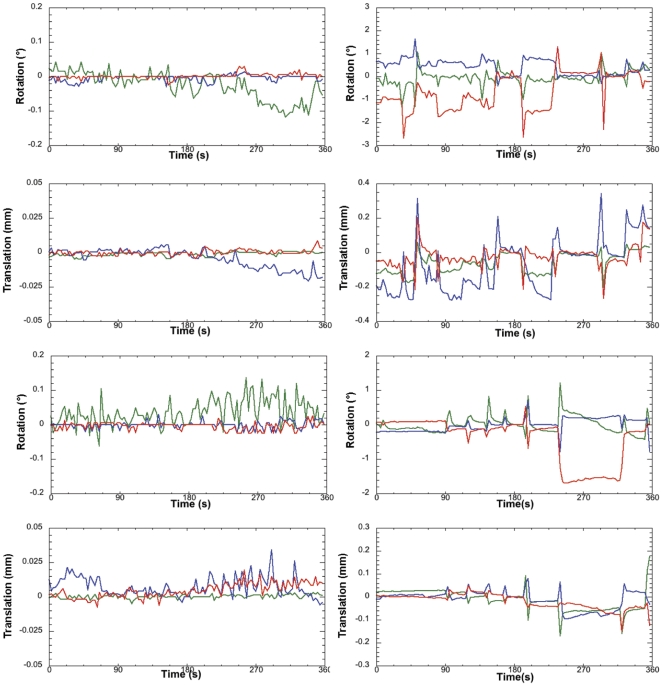
Awake Rat Motion Assessment. The motion parameters for 2 typical rats (Left column) in the study and the 2 rejected rats (Right column) are displayed. Green Line, translation/rotation X-axis, Blue Y-axis, and Red Z-axis.

Two out of 15 rats were excluded from further analysis due to excessive head motion. None were removed because of signs of stress such as vocalization. **Figure 1** depicts head motion correction parameters for 2 typical rats and for the excluded rats. In our previous study of thermal pain in other group of awake rodents [35], we demonstrated that heart rates during scanning stays within the normal range.

**Figure 2:**
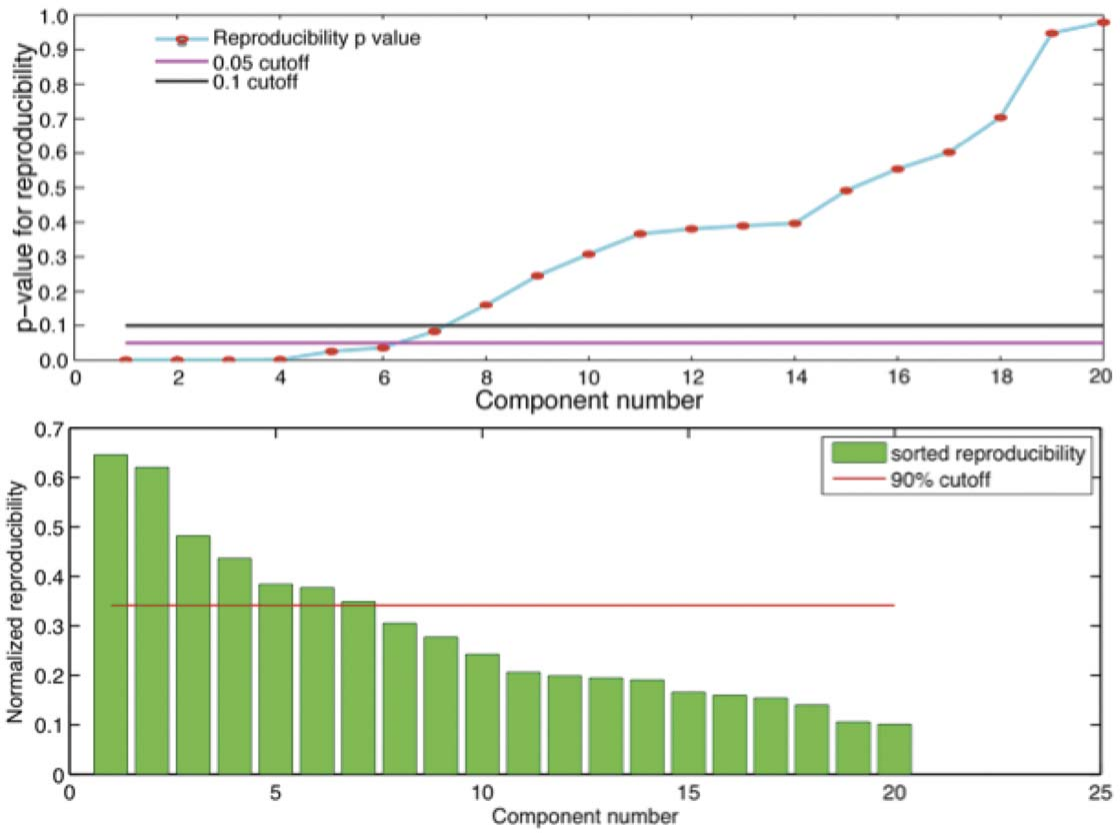
Reproducibility Analysis with RAICAR_N. The top panel displays components sorted according to reproducibility level. The red line marks the 90% cutoff as determined in the top panel. With a 90% cutoff, 7 components are above the threshold; with a 95% cutoff 6 components survive. The bottom panel depicts the normalized reproducibility and the adjusted cut-off for 90%.

**Figure 2** shows an illustrative output of the RAICAR_N analysis. The top panel shows the sorted normalized reproducibility values for each observed component. A 90% cutoff line corresponding to a p-value of 0.1 is also shown. The bottom panel depicts the normalized reproducibility with the corresponding 90% threshold line. In the present analysis, 7 components survive at a p-value of 0.1.

### Robust Reproducible Awake Rat RSNs

**Figure 3:**
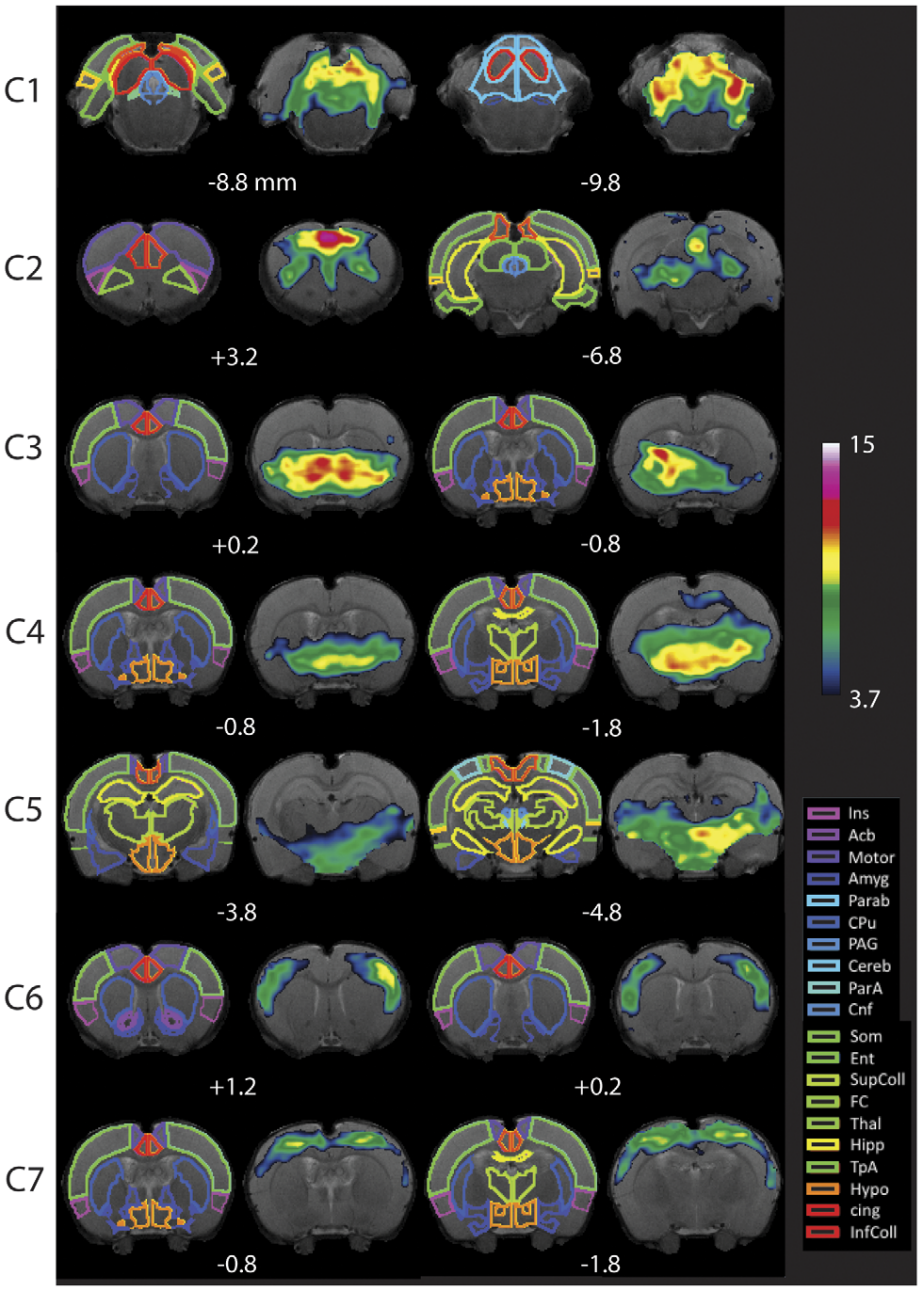
Resting State Networks in Awake Rats. Components (C1–C7) are ordered according to their reproducibility degree. Component 1 has significant cerebellar structures; Component 2 includes medial and lateral cortical structures resembling the human default mode network; Component 3 includes a basal-ganglia-hypothalamus network; Component 4 encompasses basal-ganglia-thalamus-hippocampus circuitry; Component 5 represents an autonomic pathway; Component 6 represents the sensory network; and Component 7 groups interoceptive structures to form a network. All components have been thresholded according to a mixture model approach-see Methods for details. The atlas is based on the Paxinos Atlas (Paxinos and Watson [33]). Key: Ins: Insula, AcB: Nucleus Accumbens, Motor: Motor Cortex, Amyg: Amygdala, Parab: Parabrachial, CPu: Caudate-Putamen, PAG: Periaqueductal Gray, Cereb: Cerebellum, ParA: Parietal Association Cortex, Cnf: Cuneiform nucleus, Som: Somatosensory Cortex, Ent: Entorhinal Cortex, SupColl: Superior Colliculus, FC: Frontal Cortex, Thal: Thalamus, TpA: Temporal Association Cortex, Hypo: Hypothalamus, cing: Cingulate cortex (anterior and retrosplenial), InfColl: Inferior Colliculus.

**Figure 3** depicts selected slices of the individual maps for each of the 7 components defined using RAICAR_N, for full maps see (**Figure S1**). For each component, details of structures involved in the RSN are noted in corresponding **Tables S1, S2, S3, S4, S5, S6, S7**. **Figure 3** also depicts several of the brain structures identified in the MRI images. The 7 components depict networks that can be classified according to the group of structures they include based on their level of significance of activation as well as relative size of activation (see Methods: Component Identification). These networks are noted below and are listed in order of their ranking of the RAICAR-N evaluation below.

#### Component 1 - Cerebellar Network

This network involved structures mostly in the cerebellum (cerebellar lobules 2,3,4,5) and the brainstem (periaqueductal grey (PAG) raphe nuclei), (**Table S1,** **Figure 3-C1**). Additional brain regions included the thalamus, hippocampus, cingulate cortex, olfactory cortex and insular cortex. These have been also observed in parts of ICA [27], [28] or seed-based analysis [19], [20], [26].

#### Component 2 – (Visuo-) Spatial System

The predominant regions involved are in cortical and subcortical regions with essentially only the PAG showing some brainstem involvement (**Table S2,** **Figure 3-C2**). Cortical regions included the orbitofrontal, cingulate, and retrosplenial cortices as well as insular, motor and somatosensory cortices. Similar regions were found as part of the Prefrontal Cortex network defined in awake rats by [26]. Subcortical regions included a large number of thalamic regions (anterior, ventroanterior, intralaminar and midline, and ventrolateral thalamic nuclei) and septal regions.

#### Component 3 - Basal Ganglia-Hypothalamus Network

This component is comprised mainly of subcortical structures that include components of the basal ganglia (globus pallidus, extension of the amygdala (exA), septal region, CPu, claustrum), thalamus, and hypothalamus (**Table S3 and** **Figure 3-C3**).

#### Component 4 - Basal Ganglia-Thalamus-Hippocampal Network

Regions involved in this component are shown in **Table S4** and **Figure 3-C4** and are predominantly subcortical. Based on the regions observed, including the thalamus (ventral lateral, ventral medial, ventroposterior, mediodorsal nuclei), hypothalamus, lateral globus pallidus, medial division of exA, and hippocampus.

#### Component 5 - Autonomic Network

This network is also predominantly subcortical in nature (**Table S5,** **Figure 2-C5**). As noted in the table, it encompasses mainly the hypothalamus, hippocampus (CA3), ventral tegmentum, and thalamic nuclei (VP complex, auditory, VM).

#### Component 6 – Sensory (Exteroceptive) Network

This component is essentially only cortical in nature (**Table S6,** **Figure 2-C6**). The cortical regions include primary sensory and motor cortices and cortical regions involved in auditory and olfactory processing. In addition, the orbitofrontal and insular cortices are also part of this network. Notably, this network excludes the cingulate cortex. A similar network has been reported in rats by Zhang et al. [26].

#### Component 7 - Interoceptive Network

This network includes sensory cortices as described in component 6 as well as secondary visual structures and the insular and cingulate cortices (**Table S7 and** **Figure 2-C7**).

#### Components 8–20

These components did not achieve statistical significance for robustness and reproducibility at the 90% confidence level. Inspection of them revealed that some were artifacts (9, 13, and 20 involved ventricles); 14 captured some edge of the brain; 15–19 networks seemed to already appear as part of statistically significant components. Only networks #8 and 10 reflected some biological relevant structures (CPu and cingulate/cortical ribbon, respectively). Please see Figures S2 and S3 for maps of components 8–20.

## Discussion

Here we report 7 resting state networks of a model of awake rats that are robust and reproducible. These networks were chosen statistically based on their probability of reproducibility. The networks could serve as a base to further study changes induced pharmacologically or in disease models.

### Methodological Approach

Performing awake animal imaging eliminates issues related to anesthesia [21], [36]. It might introduce other issues such as restraint-induced stress. However, this approach utilizes 3 training sessions, which has been shown to alleviate stress in the animals. King et al. have shown that stress hormone levels in the restraint rodent are close to unrestraint values after 3 trainings [37]. Our previous work in a different group of animals indicated that heart rate during scanning was within the normal range and hence taken as an indication of absence of high stress in the animal [30].

The approach utilized here allows objective identification of the most reproducible RSN components. Nevertheless, it is possible that some biologically relevant components are only moderately reproducible and, hence, this analysis might miss those components. In principle, they could be recovered by relaxing the p-value threshold on reproducibility. Furthermore, the degree of reproducibility does not necessarily imply higher biological importance.

### Awake Rat RSNs

Resting state networks represent low frequency brain fluctuations that correspond to functionally relevant networks [8], [15]. They define long distance interactions in the brain among brain substrates and are associated (in humans) with specific brain tasks at rest such as monitoring and reporting state of self, orientation, and interpretation of environment [9]. From a biological perspective, an anesthetized rat might have some of these networks' functions compromised and their measurement under anesthesia might not reflect actual brain networks [36]. For instance, one of the networks observed in awake rodents [27] is associated with integration of sensory input, cognitive processing, and output. If the rodent is unconscious, it is difficult to assess the significance of the activity of such a network, although integration might still take place because of baseline neuronal activity [36]. In an awake animal, brain function probably requires a number of “networks” that would relate to the ability to interact with the environment in terms of survival similar to that in humans. These we suggest would include (1) interoception with involvement of regions such as the insula and cingulate cortices; (2) exteroception with conscious appreciation (somatosensory cortex, thalamus) of environmental sensory cues and the ability to move toward or away (motor systems) from these cues; (3) emotional processing with the ability to evaluate emotional salience of stimuli – may include regions such as the amygdala; (4) autonomic function involving areas that include the hypothalamus; (5) general awareness – default mode network; and (6) endogenous modulation of sensory inputs (e.g., pain). It is unlikely that a single brain rhythm is associated with specific brain functions and overlap of brain regions with different components (see [38].

In our study, seven robust and reproducible components were found in awake rats. The 7 observed networks seem to be relevant and show some similarity to the networks observed in healthy humans, specifically those defined in Smith et al. [12]. We discuss these below, noted in their order of significance as noted in the results section.

#### Component 1 (Cerebellar Network)

Reported here for the first time for rats, probably was not observed in other studies because imaging did not cover the cerebellum. It is highly reproducible in rats, furthermore, this network clearly relates to component 5 of Smith et al. in humans [12], and given the role of the cerebellum it is possible to define this network as involved in action-execution and in somatosensory-perception domains (see [39]. In addition, the PAG and other brainstem regions including the raphe and pontine regions suggest a role in arousal and protective processing [40]–[42]. The network implicates a functional connectivity between the cerebellum and these brainstem regions (notably pontine in brainstem and dentate nuclei in cerebellum that are involved in input and output functions) and suggests integration and adaptation of motor and other responses [43]. It should be noted that in humans, extensive cerebellar contributions to other networks have been reported in non-motor systems [43], [44]. Brainstem regions such as the PAG, raphe nuclei, colliculi, ventral tegmental regions, and pontine reticular formation are involved in perception-somesthesis-pain domains. The network includes specific thalamic regions (mediodorsal, midline, lateral) that are involved in similar processing. In humans, similar networks involving the cerebellum have been reported and are linked to action-execution and perception-somesthesis-pain domains [12].

#### Component 2 (Spatial System)

Zhang et al. [26] detected a network based on retrosplenial seed-connectivity; in the ICA analysis of awake rodents [27], module 2 resembles our reported component 2. This network includes the retrosplenial cortex, anterior thalamic nuclei, and the hippocampus, a circuit involved in spatial memory and discriminative avoidance learning [45]. Interestingly, this component has a significant number of structures observed in the default mode network (DMN) in humans [12], including the following: cingulate, insular, motor and somatosensory, retrosplenial and orbitofrontal cortices; thalamic and septal regions in subcortical areas; and the periaqueductal gray in the brainstem. As such, we may infer that the role of this network may also relate to orientation and interpretation of the environment and detection of salient novel or familiar stimuli. However, the lack of neocortex in the rat questions if other aspects of the DMN, such as integration of visceromotor and aspects of emotion (mainly involving the ventromedial prefrontal cortex, VMPFC) and monitoring and reporting state of the self (dorsomedial prefrontal cortex, DMPFC), are taken care of by this component (although we observed inclusion of the OFC in the rat in this component as well as the cingulate and insular cortices and septal region, known to process emotions and reward). Clearly further studies are needed to properly define a DMN in rodents, perhaps with the ability to perform awake rat fMRI studies, cognitive tasks could be assessed and deactivation, commonly observed in DMN in humans [9], could be identified and associated with a rat DMN. Nevertheless, the high reproducibility of this network indicates that it plays an important role in RSNs of awake rodents.

#### Components 3 and 4 (Basal Ganglia Networks)

These include networks that encompass basal ganglia (BG, CPu, Globus Pallidus), thalamic nuclei, the amygdala, septal nucleus, and the hippocampus (see Joel and Weiner, 1994 [46]). It was also observed by Liang et al. 2011 graph analysis as part of the module 2 network related to emotion processing, and by Majeed et al. 2011 [47] in a propagation analysis of anesthetized rats. Other brain structures appeared in their study as ICA components (CPu, Hippocampus) without connections to other structures. The Basal Ganglia network of our study appeared as part of the thalamus connectivity map of the same group [26]. Hutchison detected ICA components that were confined to the thalamus, CPu, and hippocampus with no apparent connection between them. Majeed et al 2009 detected bilateral CPu network but no connection to other brain structures. It is possible that anesthesia hindered connections across these structures (as implicated by Liu et al. 2010 [36]). The thalamo-cortico-BG loops are involved in a number of integrative brain processes [48]. Their role seems to be that of action selection and reinforcement as well as perception, memory, attention, analgesia, and seizure suppression. Similar networks have been observed in humans for motor control [49], but the observed network may include a combination of other networks, such as the one involved in executive control as described by Beckmann and colleagues [11]. This subcortical network consists of structures involved in emotion (e.g., basal ganglia, extended amygdala, septal regions). The inclusion of the hypothalamus suggests that this network handles functions related to autonomic response, such as in the presence of stress. This component (4) seems complementary to component 3 in describing a network of the basal ganglia with thalamic nuclei. Thalamic involvement in resting state networks in humans has been associated with executive control [12].

#### Component 5 (Autonomic Network)

Regions such as the hypothalamus are well characterized in their role in autonomic control, but other forebrain regions are also involved [50]. It has been observed in awake rodents [27] as part of a cognitive processing module and as one of the observed ICA components. In anesthetized rats a hypothalamic network was one of 12 networks [28], although our results suggest that this network, mainly driven by the hypothalamus, also includes thalamic nuclei and the hippocampus. The hypothalamus is involved in numerous homeostatic behaviors (e.g., hunger, thirst, reproductive drive, thermoregulation, aggressive-defensive behaviors, affective-motivational tone, circadian rhythmicity/sleep-wake cycle and also immune regulation) [51]. The hippocampus in this regard may be related to defensive/stress behavior [52] and include communication with autonomic processing. The integration of thalamic regions in this network is less clear but has been observed in dysautonomia in clinical conditions such as familial dysautonomia [53]. A number of thalamic nuclei are involved in autonomic control including the paraventricular and mediodorsal nuclei. Surprisingly, cortical regions considered to be involved in autonomic control (e.g., anterior insula [54], [55] or orbitofrontal cortex [56]) were not shown to be as prominent in this network but may represent systems that have an integrative function, as shown for the hypothalamic-prefrontal connections [57]. Hypothalamic function has not only been related to classic autonomic function but is also involved in defensive behavior.

#### Component 6 (Sensory Networks-Exteroceptive and Integrative Networks)

Combined, these mainly represent sensorimotor components. This is a network commonly seen in part (bilateral S1-sparse [16], bilateral S1 [17], [21]) in addition S2 [18] and including motor and visual cortex [19], [20] or whole in most RSN studies of rats [26], [27]. In Liang's work, it was not one of the ICA components presented but it was one of the modules obtained from their graph analysis. In our results, component 6 has in addition the OFC and the insula, while component 7 includes the cingulate cortex and the insula. The presence of ACC, OFC, and insula might indicate that these components are related to a number of functions including integration of emotional, sensory and executive control. In humans these networks have been observed to split into two [11] or three [12] components. Perhaps these arise as a result of differences in the size and development of brains for both species. Baseline processing has been reported to predict stimulus-evoked responses [36]. In humans, sensorimotor and auditory networks are separate [12].

#### Component 7 (Interoceptive Networks)

This network is similar to the sensory network described above and observed in several RSN rat studies with the inclusion of insular and cingulate cortices. Liang et al. observed that one of their modules displayed some instability and further analysis resulted in a splitting of the module into 2 that encompassed the cortical ribbon observed here for components 6 and 7 [27]. Similarly to what they state, our analysis seems to indicate a complex network processing involving the sensory cortices that result in a division of the network into a sensory and interoceptive networks. Interoception has been defined as the sense of the physiological condition of the body [58]. In humans, the region most closely associated with interoception is the right anterior insula [59] as well as other cortical, such as primary and secondary somatosensory, frontal and cingulate cortices [60]. Conditions essential to survival salience are components evaluated as the interoceptive state (e.g., pain, temperature, muscular and visceral sensations, vasomotor activity, hunger, thirst). In our analysis, the insula as well as the cingulate and visual cortices were noted to be significantly involved. Interactions between the insula and cingulate have been reported in humans. Within the cingulate, two systems exist; one to the anterior and one to the posterior cingulate, that together may integrate interoception and emotional processing [61]. Here, both divisions of the rat cingulate have been observed in this network (Figure S1 and Table S7). In humans, some of the observed networks relate to the visual system with structures mostly limited to the occipital lobe [12]. In rats, we have observed limited involvement of visual related brain substrates (Component 7).

#### Components 8–20

As indicated in the results, most of them have no biological relevance, only two seem to have some biological significance and involve the CPu and the cingulate, both structures have been identified in several other networks described above. Nevertheless, these two components might reflect sub-networks within these structures that could achieve statistical significance with a larger cohort of rats.

### Caveats

#### Awake Rat Imaging

Performing studies in awake rats might induce changes in “natural” RSNs, as restraining could influence the animal's stress as well as emphasize certain networks such as environmental monitoring. As described above, stress was minimized by repeated exposure of the animals to restraint and scanner noises. Excessive (chronic) restraining, however, might be inductive of constant, elevated stress [62]. At higher magnetics fields (11.7 T) it has been shown that physiological noise may impact RSNs [63]; however, it is reasonable to expect that induced physiological noise will be accounted for by an ICA analysis.

#### Translational Equivalence

Given the obvious differences in brain morphology across species, comparisons with humans should be taken with caution. The observed networks in rodents seem to parallel those in humans. However, their assignments or interpretation should be evaluated by altering them (chemically or genetically, for example) and relating behavioral changes with RSN changes.

### Conclusions

Robust reproducible networks in awake rats were identified. Some are in agreement with those observed in other rat RSN studies. Several of the observed networks are similar to those observed in healthy humans. The elimination of the use of anesthesia might enhance significantly the opportunity to study brain alterations in well-controlled preclinical models. This approach may provide a useful addition to the use of imaging in evaluating drug effects, disease states, or genetic and strain differences.

## Supporting Information

Figure S1 **Resting State Networks in Awake Rats.** Complete maps for Components (C1–C7). All components have been thresholded according to a mixture model approach-see Methods for details. The atlas is based on the Paxinos Atlas (Paxinos and Watson). Key: Ins: Insula, AcB: Nucleus Accumbens, Motor: Motor Cortex, Amyg: Amygdala, Parab: Parabrachial, CPu: Caudate-Putamen, PAG: Periaqueductal Gray, Cereb: Cerebellum, ParA: Parietal Association Cortex, Cnf: Cuneiform nucleus, Som: Somatosensory Cortex, Ent: Entorhinal Cortex, SupColl: Superior Colliculus, FC: Frontal Cortex, Thal: Thalamus, TpA: Temporal Association Cortex, Hypo: Hypothalamus, cing: Cingulate cortex (anterior and retrosplenial), InfColl: Inferior Colliculus.
(TIF)

Figure S2 **Resting State Networks in Awake Rats.** Complete maps for Components (C8–C13). These Components did not achieve statistical significance for reproducibility. All components have been thresholded according to a mixture model approach-see Methods for details.
(TIF)

Figure S3 **Resting State Networks in Awake Rats.** Complete maps for Components (C14–C20). These Components did not achieve statistical significance for reproducibility. All components have been thresholded according to a mixture model approach-see Methods for details.
(TIF)

Table S1 **Table of Activations for Component 1.** The Table lists the most significant activated structures for the Cerebellar Network. Structures were identified using the Paxinos Atlas [33]. Structures are listed according to the fraction of the structure being active and the statistical significance of the activation (See Methods Section).
(DOCX)

Table S2 **Table of Activations for Component 2.** The Table lists the most significant activated structures for the Visuo-Spatial System. Structures were identified using the Paxinos Atlas [33]. Structures are listed according to the fraction of the structure being active and the statistical significance of the activation (See Methods Section).
(DOCX)

Table S3 **Table of Activations for Component 3.** The Table lists the most significant activated structures for the Basal Ganglia-Hypothalamus Network. Structures were identified using the Paxinos Atlas [33]. Structures are listed according to the fraction of the structure being active and the statistical significance of the activation (See Methods Section).
(DOCX)

Table S4 **Table of Activations for Component 4.** The Table lists the most significant activated structures for the Basal Ganglia-Thalamus-Hippocampus Network. Structures were identified using the Paxinos Atlas [33]. Structures are listed according to the fraction of the structure being active and the statistical significance of the activation (See Methods Section).
(DOCX)

Table S5 **Table of Activation for Component 5.** The Table lists the most significant activated structures for the Autonomic Network. Structures were identified using the Paxinos Atlas [33]. Structures are listed according to the fraction of the structure being active and the statistical significance of the activation (See Methods Section).
(DOCX)

Table S6 **Table of Activations for Component 6.** The Table lists the most significant activated structures for the Sensory Network. Structures were identified using the Paxinos Atlas [33]. Structures are listed according to the fraction of the structure being active and the statistical significance of the activation (See Methods Section).
(DOCX)

Table S7 **Table of Activation for Component 7.** The Table lists the most significant activated structures for the Interoceptive Network. Structures were identified using the Paxinos Atlas [33]. Structures are listed according to the fraction of the structure being active and the statistical significance of the activation (See Methods Section).
(DOCX)
