# Supplementary figures and images for: Robust Reproducible Resting State Networks in the Awake Rodent Brain
Source: PLoS One. 2011 Oct 18;6(10):e25701. doi: 10.1371/journal.pone.0025701 (PMC3196498; doi:10.1371/journal.pone.0025701)

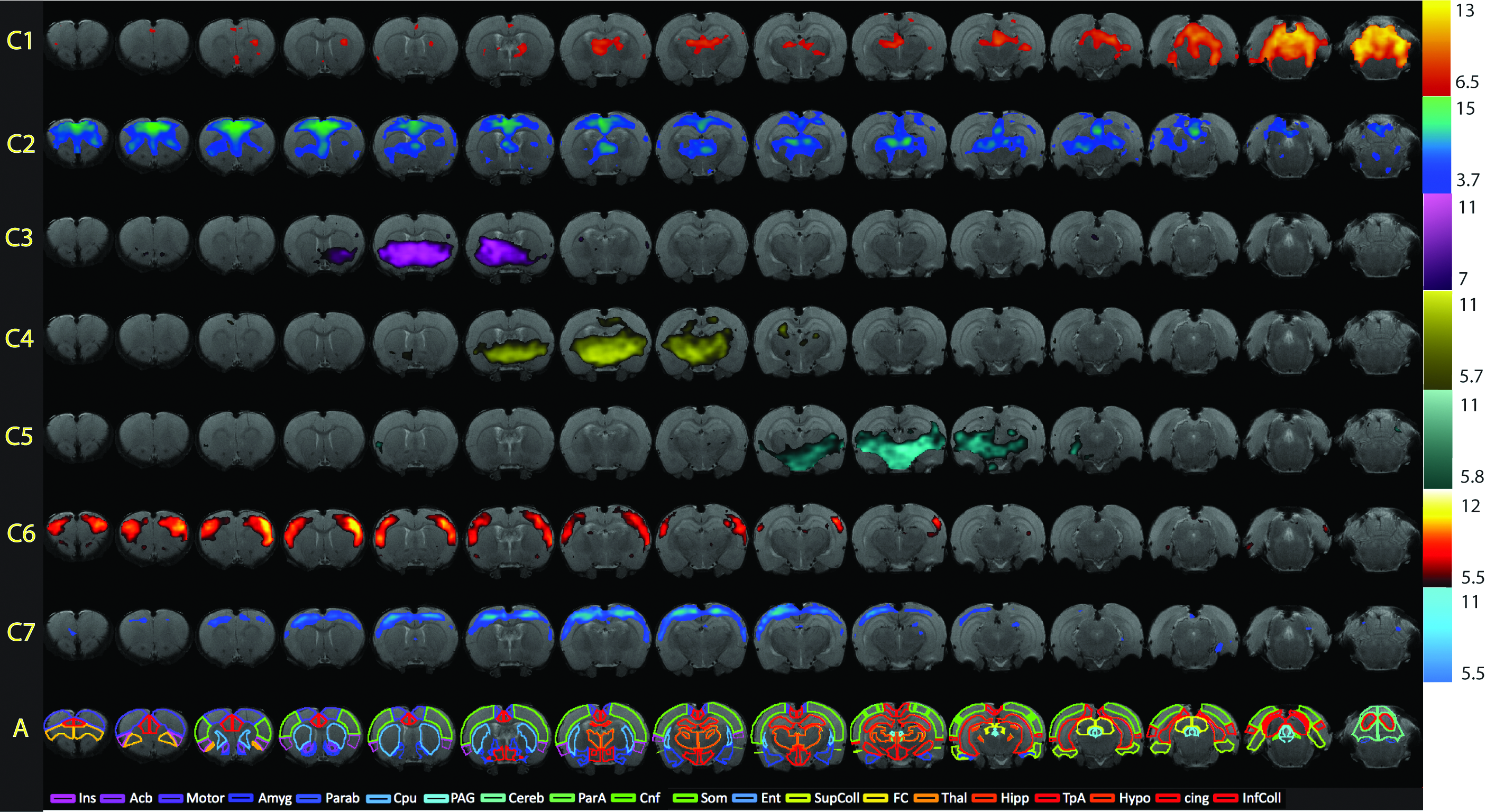

Supplement: Figure S1 — Resting State Networks in Awake Rats. Complete maps for Components (C1–C7). All components have been thresholded according to a mixture model approach-see Methods for details. The atlas is based on the Paxinos Atlas (Paxinos and Watson). Key: Ins: Insula, AcB: Nucleus Accumbens, Motor: Motor Cortex, Amyg: Amygdala, Parab: Parabrachial, CPu: Caudate-Putamen, PAG: Periaqueductal Gray, Cereb: Cerebellum, ParA: Parietal Association Cortex, Cnf: Cuneiform nucleus, Som: Somatosensory Cortex, Ent: Entorhinal Cortex, SupColl: Superior Colliculus, FC: Frontal Cortex, Thal: Thalamus, TpA: Temporal Association Cortex, Hypo: Hypothalamus, cing: Cingulate cortex (anterior and retrosplenial), InfColl: Inferior Colliculus. (TIF) [file pone.0025701.s001.tif]

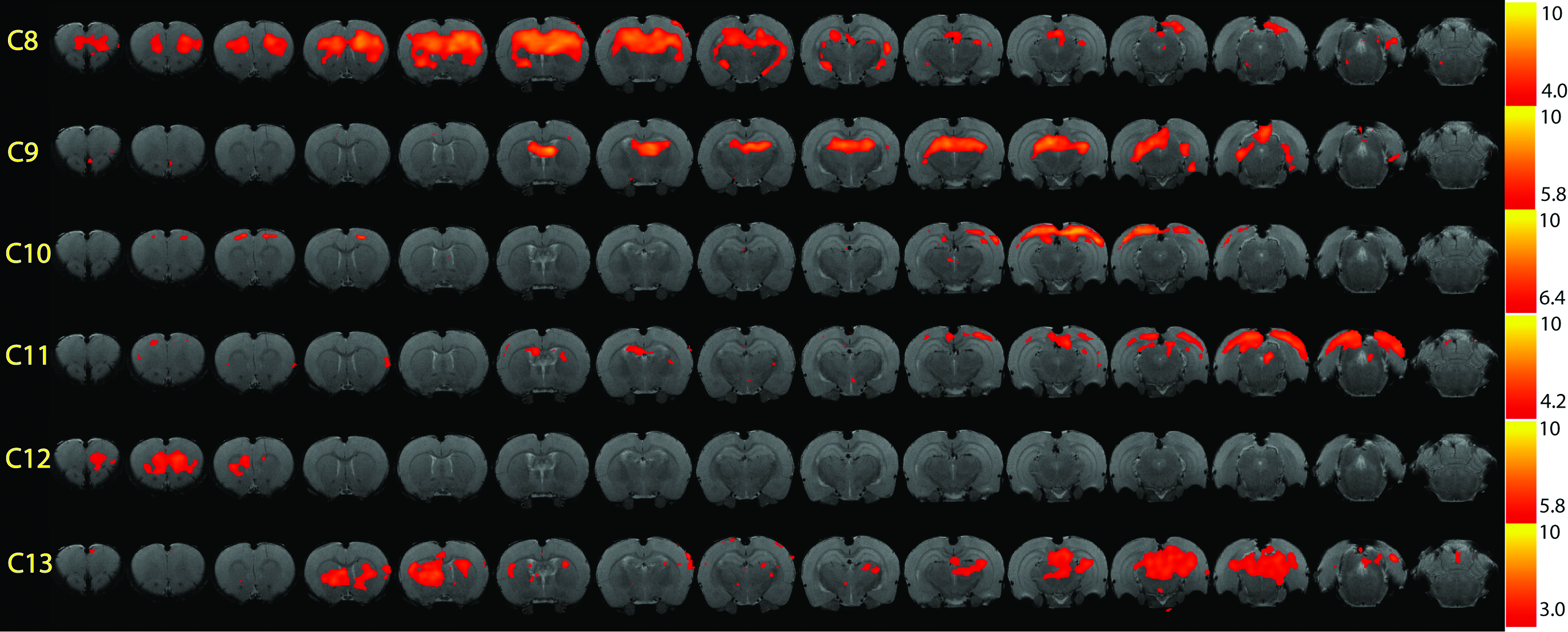

Supplement: Figure S2 — Resting State Networks in Awake Rats. Complete maps for Components (C8–C13). These Components did not achieve statistical significance for reproducibility. All components have been thresholded according to a mixture model approach-see Methods for details. (TIF) [file pone.0025701.s002.tif]

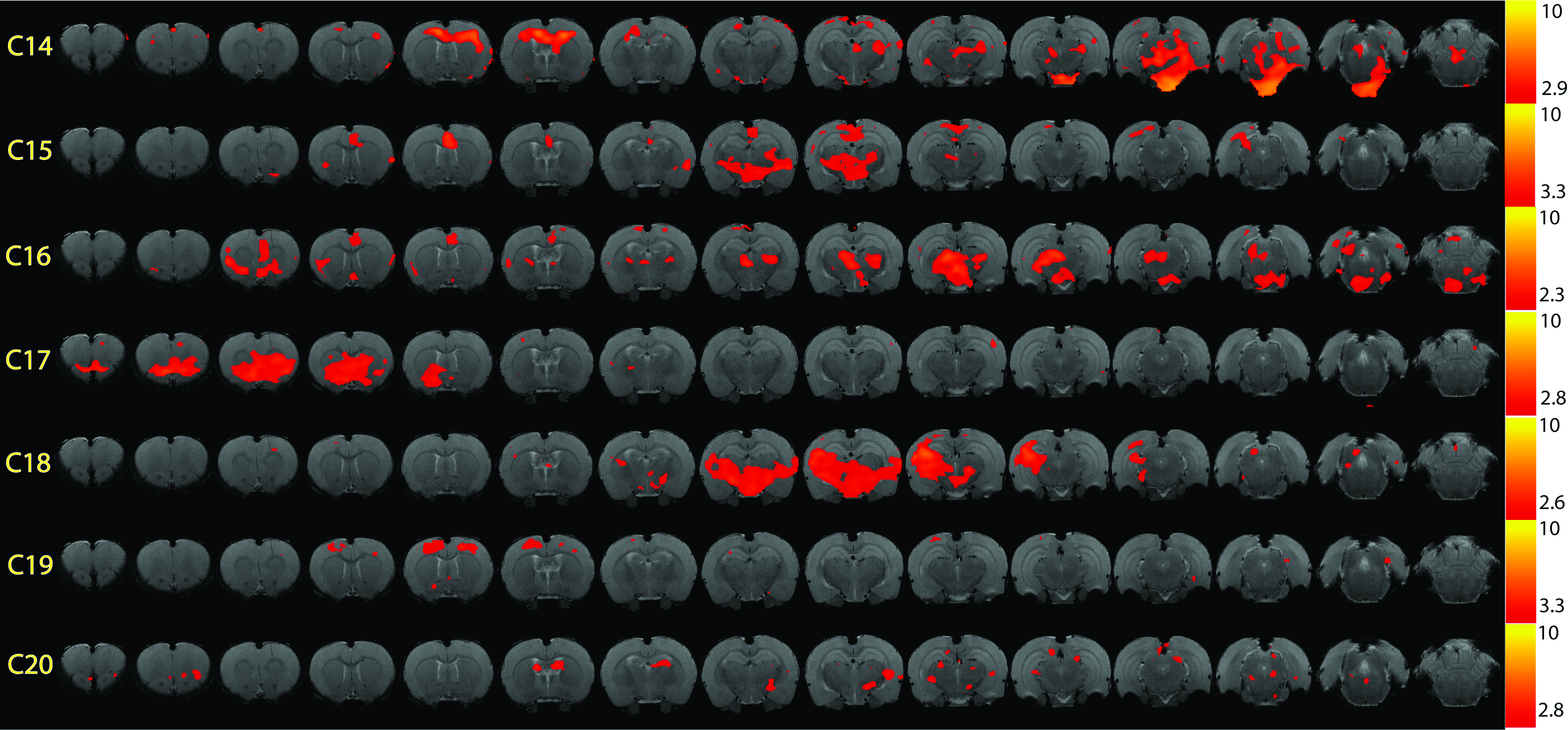

Supplement: Figure S3 — Resting State Networks in Awake Rats. Complete maps for Components (C14–C20). These Components did not achieve statistical significance for reproducibility. All components have been thresholded according to a mixture model approach-see Methods for details. (TIF) [file pone.0025701.s003.tif]
