# Supplementary material for: Robust Reproducible Resting State Networks in the Awake Rodent Brain
Source: PLoS One. 2011 Oct 18;6(10):e25701. doi: 10.1371/journal.pone.0025701 (PMC3196498; doi:10.1371/journal.pone.0025701)
Supplement: Table S1 — Table of Activations for Component 1. The Table lists the most significant activated structures for the Cerebellar Network. Structures were identified using the Paxinos Atlas [33]. Structures are listed according to the fraction of the structure being active and the statistical significance of the activation (See Methods Section). (DOCX) [file pone.0025701.s004.docx]

**Table 1: Component 1 - Cerebellar Network**

| **Brain Structure** | **Laterality** | **Active** | **Total** | **% Active** | **Avg Z** |
| --- | --- | --- | --- | --- | --- |
| Cerebellum Lobule 03 | L | 81 | 389 | 21% | 11.42 |
| Cerebellum Lobule 03 | R | 67 | 390 | 17% | 11.04 |
| Cerebellum Lobule 02 | R | 341 | 448 | 76% | 10.68 |
| Cerebellum Lobule 04 & Lobule 05 Left |  | 141 | 344 | 41% | 10.44 |
| Inferior Colliculus Left |  | 743 | 800 | 93% | 10.41 |
| Cerebellum Lobule 02 Left |  | 346 | 446 | 78% | 10.27 |
| Nucleus Parabrachialis Right |  | 34 | 34 | 100% | 10.23 |
| Inferior Colliculus Right |  | 796 | 802 | 99% | 10.20 |
| Periaqueductal Gray Ventrolateral Zone Right |  | 74 | 74 | 100% | 9.79 |
| Cerebellum Lobule 04 & Lobule 05 Right |  | 154 | 357 | 43% | 9.78 |
| Tegmental Nucleus Posterodorsal Right |  | 23 | 23 | 100% | 9.67 |
| Periaqueductal Gray Ventrolateral Zone Left |  | 79 | 79 | 100% | 9.59 |
| Central Gray of Pons Right |  | 46 | 81 | 57% | 9.33 |
| Periaqueductal Gray Dorsomedial Column Zone Left |  | 63 | 63 | 100% | 9.23 |
| Periaqueductal Gray Dorsomedial Column Zone Right |  | 63 | 63 | 100% | 9.21 |
| Nucleus Parabrachialis Left |  | 33 | 33 | 100% | 9.19 |
| Tegmental Nucleus Subpedencular Left |  | 20 | 20 | 100% | 9.09 |
| Cranial Somatic Senory Nuclei Right |  | 85 | 94 | 90% | 9.02 |
| Nucleus of Brachium of Inferior Colliculus Left |  | 36 | 36 | 100% | 8.93 |
| Tegmental Nucleus Posterodorsal Left |  | 18 | 18 | 100% | 8.88 |
| Principal Sensory Nucleus Left |  | 85 | 111 | 77% | 8.82 |
| Central Gray Alpha Part Right |  | 8 | 8 | 100% | 8.49 |
| Parabigeminal Nucleus Left |  | 9 | 9 | 100% | 8.47 |
| Cranial Somatic Senory Nuclei Left |  | 76 | 89 | 85% | 8.36 |
| Flocculus Right |  | 59 | 127 | 46% | 8.26 |
| Tegmental Nucleus Ventral Left |  | 11 | 11 | 100% | 8.25 |
| Tegmental Nucleus Ventral Right |  | 11 | 11 | 100% | 8.21 |
| Periaqueductal Gray Dorsolateral Zone Left |  | 71 | 84 | 85% | 8.14 |
| Periaqueductal Gray Precommissural Nucleus Right |  | 41 | 41 | 100% | 8.11 |
| Paraflocculus Right |  | 71 | 564 | 13% | 7.95 |
| Superior Colliculus Left |  | 708 | 821 | 86% | 7.93 |
| Periaqueductal Gray Lateral Column Zone Right |  | 112 | 146 | 77% | 7.84 |
| Vestibulocochlear Nerve Left |  | 55 | 139 | 40% | 7.84 |
| Principal Sensory Nucleus Right |  | 63 | 101 | 62% | 7.84 |
| Thalamus Anterior Nuclei Right |  | 161 | 165 | 98% | 7.83 |
| Central Gray of Pons Left |  | 46 | 74 | 62% | 7.77 |
| Periaqueductal Gray Lateral Column Zone Left |  | 96 | 136 | 71% | 7.77 |
| Parabigeminal Nucleus Right |  | 12 | 12 | 100% | 7.73 |
| Nucleus of the Lateral Lemniscus Left |  | 201 | 298 | 67% | 7.42 |
| Central Gray Alpha Part Left |  | 7 | 7 | 100% | 7.34 |
| Simple Lobule Left |  | 61 | 678 | 9% | 7.31 |
| Periaqueductal Gray Dorsolateral Zone Right |  | 49 | 88 | 56% | 7.29 |
| Raphe Nuclei Midbrain Left |  | 82 | 188 | 44% | 7.14 |
| Raphe Nuclei Midbrain Right |  | 67 | 166 | 40% | 7.02 |
| Superior Colliculus Right |  | 557 | 855 | 65% | 6.93 |
| Simple Lobule Right |  | 53 | 593 | 9% | 6.89 |
| Cerebellar Peduncle Left |  | 169 | 549 | 31% | 6.87 |
| Epithalamus Left |  | 45 | 106 | 42% | 6.87 |
| Flocculus Left |  | 51 | 112 | 46% | 6.80 |
| Nucleus of the Lateral Lemniscus Right |  | 144 | 290 | 50% | 6.80 |
| Cerebellar Peduncle Right |  | 150 | 524 | 29% | 6.59 |
| Epithalamus Right |  | 52 | 119 | 44% | 6.56 |
| Thalamus Mediodorsal Nucleus Left |  | 88 | 166 | 53% | 6.52 |
| Cranial Somatic Motor Nuclei Left |  | 37 | 142 | 26% | 6.40 |
| Pretectum Left |  | 104 | 239 | 44% | 6.39 |
| Pretectum Right |  | 127 | 228 | 56% | 6.37 |
| Paraflocculus Left |  | 55 | 503 | 11% | 6.27 |
| Auditory Thalamus Left |  | 82 | 233 | 35% | 6.09 |
| Thalamus Intralaminar Nuclei Right |  | 123 | 236 | 52% | 5.93 |
| Thalamus Anterior Nuclei Left |  | 50 | 149 | 34% | 5.91 |
| Reticular Formation Midbrian Left |  | 228 | 540 | 42% | 5.77 |
| Thalamus Lateral Nucleus Right |  | 111 | 330 | 34% | 5.54 |
| Thalamus Lateral Nucleus Left |  | 79 | 332 | 24% | 5.48 |
| Trigeminal Nerve Left |  | 117 | 612 | 19% | 5.44 |
| Thalamus Midline Nuclei Right |  | 60 | 222 | 27% | 5.42 |
| Thalamus Mediodorsal Nucleus Right |  | 44 | 162 | 27% | 5.28 |
| Thalamus Posterior Nucleus Right |  | 84 | 331 | 25% | 5.26 |
| Cranial Special Sensory Nuclei Left |  | 42 | 463 | 9% | 5.23 |
| Thalamus Posterior Nucleus Left |  | 48 | 337 | 14% | 4.97 |
| Reticular Formation Midbrian Right |  | 131 | 547 | 24% | 4.90 |
| Hippocampal Formation Subicular Complex Left |  | 250 | 807 | 31% | 4.86 |
| Olfactory Cortex Lateral Right |  | 268 | 3351 | 8% | 4.74 |
| Olfactory Cortex Lateral Left |  | 308 | 3380 | 9% | 4.63 |
| Trigeminal Nerve Right |  | 104 | 609 | 17% | 4.58 |
| Reticular Thalamic Nucleus Left |  | 66 | 269 | 25% | 4.56 |
| Thalamus Ventral Posterior Complex Left |  | 41 | 415 | 10% | 4.54 |
| Thalamus Intralaminar Nuclei Left |  | 50 | 243 | 21% | 4.42 |
| Fimbria Fronix Left |  | 56 | 604 | 9% | 4.24 |
| Hippocampal Formation Dentate Gyrus Left |  | 115 | 890 | 13% | 4.01 |
| Corticospinal Tract Left |  | 58 | 825 | 7% | 3.99 |
| Reticular Formation Pontomedullary Right |  | 38 | 523 | 7% | 3.97 |
| Hippocampal Formation CA3 Field Left |  | 87 | 725 | 12% | 3.89 |
| Perirhinal Cortex Left |  | 67 | 730 | 9% | 3.77 |
| Retrosplenial Cortex Left |  | 155 | 934 | 17% | 3.75 |
| Cingulate Cortex Right |  | 36 | 994 | 4% | 3.75 |
| Cingulate Cortex Left |  | 51 | 953 | 5% | 3.52 |
| Reticular Formation Pontomedullary Left |  | 54 | 523 | 10% | 3.42 |
| Visual Cortex Secondary Left |  | 53 | 1244 | 4% | 3.42 |
| Striatum Dorsal Left |  | 137 | 2939 | 5% | 3.19 |
| Retrosplenial Cortex Right |  | 109 | 1134 | 10% | 3.17 |
| Corpus Callosum Left |  | 71 | 1892 | 4% | 3.04 |
| Hippocampal Formation Subicular Complex Right |  | 56 | 823 | 7% | 3.03 |
| Extended Amygdala Medial Division Left |  | 37 | 490 | 8% | 2.79 |
| Insular Cortex Left |  | 50 | 1259 | 4% | 2.50 |
| Insular Cortex Right |  | 40 | 1228 | 3% | 2.47 |
