# Supplementary material for: Robust Reproducible Resting State Networks in the Awake Rodent Brain
Source: PLoS One. 2011 Oct 18;6(10):e25701. doi: 10.1371/journal.pone.0025701 (PMC3196498; doi:10.1371/journal.pone.0025701)
Supplement: Table S2 — Table of Activations for Component 2. The Table lists the most significant activated structures for the Visuo-Spatial System. Structures were identified using the Paxinos Atlas [33]. Structures are listed according to the fraction of the structure being active and the statistical significance of the activation (See Methods Section). (DOCX) [file pone.0025701.s005.docx]

**Table 2: Component 2 – Visuo-Spatial Network**

| **Brain Structure** | **Active** | **Total** | **% Active** | **Avg Z** |
| --- | --- | --- | --- | --- |
| Periaqueductal Gray Precommissural Nucleus Right | 41 | 41 | 100% | 9.58 |
| Periaqueductal Gray Precommissural Nucleus Left | 41 | 41 | 100% | 9.42 |
| Cingulate Cortex Right | 985 | 994 | 99% | 9.40 |
| Cingulate Cortex Left | 947 | 953 | 99% | 9.19 |
| Motor Cortex Secondary Left | 984 | 1278 | 77% | 8.87 |
| Magnocellular Nucleus of the Posterior Commissure Right | 14 | 14 | 100% | 8.80 |
| Motor Cortex Secondary Right | 1020 | 1249 | 82% | 8.80 |
| Thalamus Anterior Nuclei Left | 103 | 149 | 69% | 8.48 |
| Septal Region Posterior Group Right | 58 | 58 | 100% | 7.72 |
| Magnocellular Nucleus of the Posterior Commissure Left | 15 | 15 | 100% | 7.56 |
| Epithalamus Right | 114 | 119 | 96% | 7.50 |
| Somatosensory Cortex Primary Hindlimb Region Right | 286 | 309 | 93% | 7.47 |
| Epithalamus Left | 94 | 106 | 89% | 7.46 |
| Thalamus Anterior Nuclei Right | 119 | 165 | 72% | 7.30 |
| Thalamus Vental Lateral & Ventral Anterior Nuclei Left | 93 | 98 | 95% | 7.28 |
| Pretectum Right | 182 | 228 | 80% | 7.18 |
| Motor Cortex Primary Right | 1222 | 1643 | 74% | 7.14 |
| Thalamus Intralaminar Nuclei Left | 235 | 243 | 97% | 7.00 |
| Thalamus Midline Nuclei Right | 172 | 222 | 77% | 6.94 |
| Thalamus Intralaminar Nuclei Right | 234 | 236 | 99% | 6.92 |
| Motor Cortex Primary Left | 1063 | 1830 | 58% | 6.86 |
| Cerebellum Lobule 03 Right | 47 | 390 | 12% | 6.70 |
| Pretectum Left | 207 | 239 | 87% | 6.70 |
| Olfactory Cortex Medial Right | 244 | 662 | 37% | 6.66 |
| Somatosensory Cortex Primary Hindlimb Region Left | 250 | 304 | 82% | 6.61 |
| Auditory Thalamus Right | 232 | 235 | 99% | 6.58 |
| Thalamus Midline Nuclei Left | 160 | 209 | 77% | 6.57 |
| Septal Region Lateral Group Right | 396 | 448 | 88% | 6.53 |
| Septal Region Medial Group Right | 49 | 185 | 26% | 6.51 |
| Thalamus Nucleus Submedius Left | 29 | 29 | 100% | 6.47 |
| Orbitofrontal Cortex Left | 585 | 1142 | 51% | 6.47 |
| Retrosplenial Cortex Left | 694 | 934 | 74% | 6.42 |
| Periaqueductal Gray Dorsomedial Column Zone Left | 62 | 63 | 98% | 6.41 |
| Somatosensory Cortex Primary Forelimb Region Right | 477 | 632 | 75% | 6.38 |
| Superior Colliculus Left | 614 | 821 | 75% | 6.36 |
| Thalamus Lateral Nucleus Right | 237 | 330 | 72% | 6.34 |
| Reticular Formation Midbrian Right | 285 | 547 | 52% | 6.29 |
| Superior Colliculus Right | 549 | 855 | 64% | 6.29 |
| Thalamus Mediodorsal Nucleus Right | 162 | 162 | 100% | 6.27 |
| Septal Region Lateral Group Left | 348 | 489 | 71% | 6.23 |
| Thalamus Vental Lateral & Ventral Anterior Nuclei Right | 87 | 91 | 96% | 6.20 |
| Retrosplenial Cortex Right | 739 | 1134 | 65% | 6.17 |
| Thalamus Mediodorsal Nucleus Left | 161 | 166 | 97% | 6.15 |
| Insular Cortex Right | 315 | 1228 | 26% | 6.15 |
| Periaqueductal Gray Lateral Column Zone Right | 81 | 146 | 55% | 6.14 |
| Rubral Area Right | 145 | 274 | 53% | 6.12 |
| Orbitofrontal Cortex Right | 599 | 1092 | 55% | 6.08 |
| Claustrum Right | 106 | 142 | 75% | 6.05 |
| Interstitial Nucleus of the Medial Longitudinal Fasciculus Right | 61 | 70 | 87% | 6.01 |
| Olfactory Cortex Medial Left | 227 | 695 | 33% | 5.97 |
| Thalamus Posterior Nucleus Right | 276 | 331 | 83% | 5.93 |
| Auditory Thalamus Left | 214 | 233 | 92% | 5.87 |
| Cingulum Right | 108 | 154 | 70% | 5.85 |
| Cingulum Left | 121 | 155 | 78% | 5.85 |
| Darkschewitch Nucleus Right | 12 | 12 | 100% | 5.82 |
| Inferior Colliculus Right | 373 | 802 | 47% | 5.80 |
| Olfactory Nucleus Anterior Right | 34 | 894 | 4% | 5.78 |
| Claustrum Left | 56 | 143 | 39% | 5.76 |
| Cerebellum Lobule 03 Left | 62 | 389 | 16% | 5.74 |
| Thalamus Lateral Nucleus Left | 164 | 332 | 49% | 5.68 |
| Somatosensory Cortex Primary Trunk Region Right | 263 | 473 | 56% | 5.65 |
| Somatosensory Cortex Primary Forelimb Region Left | 358 | 644 | 56% | 5.60 |
| Corpus Callosum Right | 800 | 1863 | 43% | 5.60 |
| Reticular Formation Midbrian Left | 116 | 540 | 21% | 5.47 |
| Somatosensory Cortex Primary Trunk Region Left | 262 | 458 | 57% | 5.40 |
| Corpus Callosum Left | 825 | 1892 | 44% | 5.40 |
| Thalamus Posterior Nucleus Left | 275 | 337 | 82% | 5.34 |
| Rubral Area Left | 44 | 271 | 16% | 5.34 |
| Hippocampal Formation Subicular Complex Left | 100 | 807 | 12% | 5.32 |
| Optic Nerve Left | 70 | 225 | 31% | 5.31 |
| Extended Amygdala Central Division Right | 71 | 456 | 16% | 5.26 |
| Auditory Radiation Left | 28 | 28 | 100% | 5.26 |
| Septal Region Posterior Group Left | 52 | 65 | 80% | 5.23 |
| Somatosensory Cortex Primary Dysgranular Region Left | 108 | 232 | 47% | 5.22 |
| Somatosensory Cortex Primary Dysgranular Region Right | 144 | 233 | 62% | 5.18 |
| Periaqueductal Gray Dorsomedial Column Zone Right | 61 | 63 | 97% | 5.16 |
| Insular Cortex Left | 341 | 1259 | 27% | 5.15 |
| Periaqueductal Gray Dorsolateral Zone Left | 56 | 84 | 67% | 5.12 |
| Fimbria Fronix Left | 152 | 604 | 25% | 5.10 |
| Periaqueductal Gray Lateral Column Zone Left | 67 | 136 | 49% | 5.05 |
| Auditory Radiation Right | 29 | 29 | 100% | 5.05 |
| Septal Region Medial Group Left | 55 | 160 | 34% | 5.04 |
| Tegmental Area Dorsomedial Right | 37 | 40 | 93% | 4.97 |
| Somatosensory Cortex Primary Barrel Field Left | 405 | 1590 | 25% | 4.97 |
| Inferior Colliculus Left | 258 | 800 | 32% | 4.95 |
| Thalamus Lateral Geniculate Nucleus Left | 114 | 179 | 64% | 4.95 |
| Hippocampal Formation Subicular Complex Right | 342 | 823 | 42% | 4.91 |
| Thalamus Ventral Medial Nucleus Left | 59 | 109 | 54% | 4.89 |
| Olfactory Nucleus Anterior Left | 59 | 874 | 7% | 4.86 |
| Thalamus Ventral Medial Nucleus Right | 77 | 105 | 73% | 4.86 |
| Hippocampal Formation Dentate Gyrus Right | 247 | 892 | 28% | 4.84 |
| Hippocampal Formation CA1 Field Right | 95 | 973 | 10% | 4.82 |
| Striatum Dorsal Right | 869 | 2932 | 30% | 4.82 |
| Somatosensory Cortex Primary Jaw Region Right | 170 | 688 | 25% | 4.79 |
| Thalamus Ventral Posterior Complex Right | 322 | 417 | 77% | 4.77 |
| Periaqueductal Gray Dorsolateral Zone Right | 48 | 88 | 55% | 4.76 |
| Striatum Ventral Right | 56 | 248 | 23% | 4.76 |
| Fimbria Fronix Right | 78 | 550 | 14% | 4.70 |
| Temporal Association Cortex Right | 118 | 492 | 24% | 4.70 |
| Thalamus Ventral Posterior Complex Left | 222 | 415 | 53% | 4.68 |
| Reticular Thalamic Nucleus Right | 109 | 253 | 43% | 4.68 |
| Striatum Dorsal Left | 409 | 2939 | 14% | 4.67 |
| Somatosensory Cortex Primary Barrel Field Right | 254 | 1600 | 16% | 4.62 |
| Parietal Cortex Posterior Area Left | 125 | 290 | 43% | 4.59 |
| Hippocampal Formation CA1 Field Left | 161 | 984 | 16% | 4.59 |
| Somatosensory Cortex Secondary Left | 228 | 918 | 25% | 4.57 |
| Hippocampal Formation CA3 Field Left | 138 | 725 | 19% | 4.57 |
| Somatosensory Cortex Primary Jaw Region Left | 69 | 629 | 11% | 4.54 |
| Hippocampal Formation Dentate Gyrus Left | 177 | 890 | 20% | 4.53 |
| Reticular Formation Pontomedullary Left | 66 | 523 | 13% | 4.53 |
| Somatosensory Cortex Primary Upper Lip Region Left | 121 | 466 | 26% | 4.46 |
| Hypothalamus Lateral Zone Right | 83 | 602 | 14% | 4.43 |
| Auditory Cortex Secondary Left | 59 | 397 | 15% | 4.38 |
| Extended Amygdala Medial Division Right | 80 | 491 | 16% | 4.31 |
| Intercollicular Nucleus Right | 34 | 41 | 83% | 4.31 |
| Hippocampal Formation CA3 Field Right | 56 | 678 | 8% | 4.31 |
| Somatosensory Cortex Primary Left | 61 | 381 | 16% | 4.29 |
| Reticular Thalamic Nucleus Left | 39 | 269 | 14% | 4.29 |
| Visual Cortex Secondary Right | 356 | 1312 | 27% | 4.25 |
| Somatosensory Cortex Secondary Right | 86 | 887 | 10% | 4.23 |
| Thalamus Lateral Geniculate Nucleus Right | 55 | 161 | 34% | 4.15 |
| Perirhinal Cortex Right | 63 | 759 | 8% | 4.13 |
| Visual Cortex Primary Right | 131 | 856 | 15% | 4.05 |
| Cerebellum Lobule 02 Left | 53 | 446 | 12% | 4.04 |
| Visual Cortex Secondary Left | 236 | 1244 | 19% | 4.01 |
| Zona incerta Right | 84 | 250 | 34% | 3.94 |
| Olfactory Cortex Lateral Left | 198 | 3380 | 6% | 3.91 |
| Reticular Formation Pontomedullary Right | 39 | 523 | 7% | 3.83 |
| Auditory Cortex Primary Right | 48 | 623 | 8% | 3.83 |
| Somatosensory Cortex Primary Upper Lip Region Right | 57 | 467 | 12% | 3.78 |
| Visual Cortex Primary Left | 66 | 778 | 8% | 3.54 |
| Corticospinal Tract Right | 34 | 811 | 4% | 3.52 |
