# Supplementary material for: Robust Reproducible Resting State Networks in the Awake Rodent Brain
Source: PLoS One. 2011 Oct 18;6(10):e25701. doi: 10.1371/journal.pone.0025701 (PMC3196498; doi:10.1371/journal.pone.0025701)
Supplement: Table S3 — Table of Activations for Component 3. The Table lists the most significant activated structures for the Basal Ganglia-Hypothalamus Network. Structures were identified using the Paxinos Atlas [33]. Structures are listed according to the fraction of the structure being active and the statistical significance of the activation (See Methods Section). (DOCX) [file pone.0025701.s006.docx]

**Table 3: Component 3 - Basal Ganglia/Hypothalamus Network**

| **Brain Structure** | **Active** | **Total** | **% Active** | **Avg Z** |
| --- | --- | --- | --- | --- |
| Globus Pallidus Lateral Right | 151 | 368 | 41% | 9.60 |
| Extended Amygdala Medial Division Right | 200 | 491 | 41% | 9.55 |
| Fimbria Fronix Right | 34 | 550 | 6% | 9.48 |
| Septal Region Lateral Group Right | 227 | 448 | 51% | 9.42 |
| Extended Amygdala Central Division Right | 127 | 456 | 28% | 9.33 |
| Perifornica Nucleus Anterior Right | 8 | 8 | 100% | 9.22 |
| Septal Region Medial Group Right | 155 | 185 | 84% | 9.19 |
| Septal Region Medial Group Left | 126 | 160 | 79% | 9.19 |
| Anterior Commissure Left | 34 | 120 | 28% | 9.18 |
| Septal Region Lateral Group Left | 200 | 489 | 41% | 9.10 |
| Striatum Dorsal Right | 1205 | 2932 | 41% | 9.09 |
| Perifornica Nucleus Anterior Left | 11 | 11 | 100% | 9.01 |
| Hypothalamus Periventicular Zone Left | 38 | 102 | 37% | 8.96 |
| Striatum Dorsal Left | 761 | 2939 | 26% | 8.94 |
| Ventral Pallidum Right | 153 | 243 | 63% | 8.93 |
| Fimbria Fronix Left | 36 | 604 | 6% | 8.90 |
| Extended Amygdala Medial Division Left | 208 | 490 | 42% | 8.86 |
| Claustrum Right | 31 | 142 | 22% | 8.84 |
| Ventral Pallidum Left | 166 | 244 | 68% | 8.47 |
| Insular Cortex Right | 136 | 1228 | 11% | 8.46 |
| Hypothalamus Lateral Zone Right | 63 | 602 | 10% | 8.27 |
| Hypothalamus Medial Zone Left | 63 | 577 | 11% | 8.26 |
| Striatum Lateral Stripe Right | 12 | 12 | 100% | 8.25 |
| Extended Amygdala Central Division Left | 252 | 471 | 54% | 8.23 |
| Hypothalamus Medial Zone Right | 53 | 566 | 9% | 8.22 |
| Olfactory Cortex Lateral Left | 176 | 3380 | 5% | 8.21 |
| Hypothalamus Lateral Zone Left | 65 | 627 | 10% | 8.19 |
| Striatum Ventral Left | 140 | 253 | 55% | 8.13 |
| Septal Region Posterior Group Right | 33 | 58 | 57% | 8.09 |
| Insular Cortex Left | 163 | 1259 | 13% | 7.99 |
| Globus Pallidus Lateral Left | 62 | 382 | 16% | 7.91 |
| Olfactory Cortex Lateral Right | 112 | 3351 | 3% | 7.90 |
| Somatosensory Cortex Secondary Left | 80 | 918 | 9% | 7.66 |
| Somatosensory C Primary Upper Lip Region Left | 45 | 466 | 10% | 7.55 |
