# Supplementary material for: Robust Reproducible Resting State Networks in the Awake Rodent Brain
Source: PLoS One. 2011 Oct 18;6(10):e25701. doi: 10.1371/journal.pone.0025701 (PMC3196498; doi:10.1371/journal.pone.0025701)
Supplement: Table S4 — Table of Activations for Component 4. The Table lists the most significant activated structures for the Basal Ganglia-Thalamus-Hippocampus Network. Structures were identified using the Paxinos Atlas [33]. Structures are listed according to the fraction of the structure being active and the statistical significance of the activation (See Methods Section). (DOCX) [file pone.0025701.s007.docx]

**Table 4: Component 4 - Basal Ganglia/Thalamus/Hippocampal Network**

| **Brain Structure** | **Active** | **Total** | **% Active** | **Avg Z** |
| --- | --- | --- | --- | --- |
| Thalamus Ventral Medial Nucleus Right | 61 | 105 | 58% | 9.09 |
| Hypothalamus Periventicular Zone Right | 54 | 108 | 50% | 9.06 |
| Hypothalamus Periventicular Zone Left | 56 | 102 | 55% | 8.88 |
| Zona incerta Right | 69 | 250 | 28% | 8.61 |
| Thalamus Nucleus Submedius Right | 34 | 34 | 100% | 8.52 |
| Perifornica Nucleus Anterior Right | 8 | 8 | 100% | 8.46 |
| Globus Pallidus Medial Right | 34 | 34 | 100% | 8.42 |
| Thalamus Vental Lateral & Ventral Anterior Nuclei Right | 91 | 91 | 100% | 8.41 |
| Thalamus Midline Nuclei Right | 144 | 222 | 65% | 8.38 |
| Reticular Thalamic Nucleus Right | 195 | 253 | 77% | 8.28 |
| Thalamus Nucleus Submedius Left | 29 | 29 | 100% | 8.27 |
| Perifornica Nucleus Anterior Left | 11 | 11 | 100% | 8.23 |
| Hypothalamus Lateral Zone Right | 262 | 602 | 44% | 8.19 |
| Thalamus Midline Nuclei Left | 149 | 209 | 71% | 8.17 |
| Thalamus Ventral Medial Nucleus Left | 61 | 109 | 56% | 8.16 |
| Reticular Thalamic Nucleus Left | 206 | 269 | 77% | 8.13 |
| Thalamus Vental Lateral & Ventral Anterior Nuclei Left | 98 | 98 | 100% | 8.13 |
| Extended Amygdala Medial Division Left | 253 | 490 | 52% | 8.13 |
| Thalamus Anterior Nuclei Left | 149 | 149 | 100% | 8.12 |
| Extended Amygdala Medial Division Right | 272 | 491 | 55% | 8.04 |
| Globus Pallidus Lateral Left | 347 | 382 | 91% | 8.03 |
| Globus Pallidus Lateral Right | 341 | 368 | 93% | 7.91 |
| Thalamus Anterior Nuclei Right | 165 | 165 | 100% | 7.88 |
| Hypothalamus Lateral Zone Left | 264 | 627 | 42% | 7.88 |
| Thalamus Ventral Posterior Complex Left | 158 | 415 | 38% | 7.86 |
| Thalamus Ventral Posterior Complex Right | 156 | 417 | 37% | 7.79 |
| Hypothalamus Medial Zone Right | 350 | 566 | 62% | 7.73 |
| Extended Amygdala Central Division Left | 152 | 471 | 32% | 7.65 |
| Ventral Pallidum Left | 46 | 244 | 19% | 7.53 |
| Zona incerta Left | 72 | 261 | 28% | 7.51 |
| Extended Amygdala Central Division Right | 171 | 456 | 38% | 7.50 |
| Thalamus Intralaminar Nuclei Left | 102 | 243 | 42% | 7.50 |
| Hypothalamus Medial Zone Left | 284 | 577 | 49% | 7.41 |
| Striatum Dorsal Left | 652 | 2939 | 22% | 7.40 |
| Thalamus Posterior Nucleus Left | 108 | 337 | 32% | 7.37 |
| Globus Pallidus Medial Left | 38 | 38 | 100% | 7.31 |
| Thalamus Intralaminar Nuclei Right | 103 | 236 | 44% | 7.29 |
| Thalamus Posterior Nucleus Right | 75 | 331 | 23% | 7.25 |
| Fimbria Fronix Right | 104 | 550 | 19% | 7.14 |
| Angular Thalamic Nucleus Left | 21 | 21 | 100% | 7.10 |
| Insular Cortex Left | 192 | 1259 | 15% | 7.08 |
| Angular Thalamic Nucleus Right | 17 | 17 | 100% | 7.03 |
| Fimbria Fronix Left | 125 | 604 | 21% | 6.96 |
| Somatosensory Cortex Secondary Left | 200 | 918 | 22% | 6.91 |
| Ventral Pallidum Right | 59 | 243 | 24% | 6.87 |
| Thalamus Mediodorsal Nucleus Left | 125 | 166 | 75% | 6.83 |
| Thalamus Mediodorsal Nucleus Right | 77 | 162 | 48% | 6.82 |
| Somatosensory Cortex Primary Hindlimb Region Left | 45 | 304 | 15% | 6.80 |
| Striatum Dorsal Right | 579 | 2932 | 20% | 6.77 |
| Hippocampal Formation Dentate Gyrus Right | 69 | 892 | 8% | 6.73 |
| Hippocampal Formation CA2 Field Right | 86 | 317 | 27% | 6.71 |
| Thalamus Lateral Nucleus Left | 107 | 332 | 32% | 6.62 |
| Epithalamus Right | 31 | 119 | 26% | 6.62 |
| Thalamus Lateral Nucleus Right | 166 | 330 | 50% | 6.60 |
| Insular Cortex Right | 116 | 1228 | 9% | 6.58 |
| Hippocampal Formation CA3 Field Right | 81 | 678 | 12% | 6.56 |
| Corpus Callosum Left | 138 | 1892 | 7% | 6.42 |
| Cingulum Left | 48 | 155 | 31% | 6.41 |
| Retrosplenial Cortex Left | 91 | 934 | 10% | 6.33 |
| Corpus Callosum Right | 56 | 1863 | 3% | 6.22 |
| Hippocampal Formation CA1 Field Right | 39 | 973 | 4% | 6.16 |
| Retrosplenial Cortex Right | 82 | 1134 | 7% | 6.15 |
| Somatosensory Cortex Primary Forelimb Region Left | 35 | 644 | 5% | 6.13 |
| Somatosensory Cortex Secondary Right | 41 | 887 | 5% | 6.10 |
| Motor Cortex Secondary Right | 30 | 1249 | 2% | 6.05 |
