# Supplementary material for: Robust Reproducible Resting State Networks in the Awake Rodent Brain
Source: PLoS One. 2011 Oct 18;6(10):e25701. doi: 10.1371/journal.pone.0025701 (PMC3196498; doi:10.1371/journal.pone.0025701)
Supplement: Table S5 — Table of Activation for Component 5. The Table lists the most significant activated structures for the Autonomic Network. Structures were identified using the Paxinos Atlas [33]. Structures are listed according to the fraction of the structure being active and the statistical significance of the activation (See Methods Section). (DOCX) [file pone.0025701.s008.docx]

**Table 5: Component 5 - Autonomic Network**

| **Brain Structure** | **Active** | **Total** | **% Active** | **Avg Z** |
| --- | --- | --- | --- | --- |
| Auditory Radiation Left | 28 | 28 | 100% | 9.10 |
| Zona incerta Left | 189 | 261 | 72% | 9.02 |
| Hypothalamus Lateral Zone Left | 307 | 627 | 49% | 8.62 |
| Hypothalamus Periventicular Zone Right | 41 | 108 | 38% | 8.39 |
| Thalamus Lateral Geniculate Nucleus Left | 102 | 179 | 57% | 8.38 |
| Hypothalamus Periventicular Zone Left | 35 | 102 | 34% | 8.34 |
| Hypothalamus Lateral Zone Right | 305 | 602 | 51% | 8.22 |
| Hypothalamus Medial Zone Right | 153 | 566 | 27% | 8.17 |
| Dorsal Column Left | 47 | 125 | 38% | 8.17 |
| Hypothalamus Medial Zone Left | 183 | 577 | 32% | 8.16 |
| Corticospinal Tract Left | 254 | 825 | 31% | 8.00 |
| Rubral Area Left | 123 | 271 | 45% | 7.95 |
| Auditory Thalamus Right | 200 | 235 | 85% | 7.93 |
| Zona incerta Right | 165 | 250 | 66% | 7.89 |
| Temporal Association Cortex Left | 33 | 462 | 7% | 7.84 |
| Rubral Area Right | 160 | 274 | 58% | 7.84 |
| Hippocampal Formation Dentate Gyrus Right | 139 | 892 | 16% | 7.81 |
| Fimbria Fronix Right | 143 | 550 | 26% | 7.80 |
| Thalamus Ventral Posterior Complex Left | 212 | 415 | 51% | 7.78 |
| Hippocampal Formation CA3 Field Right | 362 | 678 | 53% | 7.76 |
| Hippocampal Formation CA3 Field Left | 235 | 725 | 32% | 7.72 |
| Dorsal Column Right | 53 | 122 | 43% | 7.72 |
| Fimbria Fronix Left | 147 | 604 | 24% | 7.71 |
| Subthalamic Nucleus Left | 23 | 23 | 100% | 7.70 |
| Auditory Radiation Right | 29 | 29 | 100% | 7.69 |
| Tegmental Area Ventral Right | 91 | 128 | 71% | 7.60 |
| Thalamus Posterior Nucleus Right | 136 | 331 | 41% | 7.60 |
| Tegmental Area Ventral Left | 90 | 131 | 69% | 7.59 |
| Thalamus Ventral Posterior Complex Right | 75 | 417 | 18% | 7.55 |
| Substantia Nigra Right | 150 | 176 | 85% | 7.48 |
| Optic Nuclei Accessory Right | 12 | 12 | 100% | 7.44 |
| Hippocampal Formation Subicular Complex Right | 69 | 823 | 8% | 7.39 |
| Corpus Callosum Right | 62 | 1863 | 3% | 7.39 |
| Thalamus Lateral Geniculate Nucleus Right | 104 | 161 | 65% | 7.37 |
| Auditory Thalamus Left | 103 | 233 | 44% | 7.28 |
| Temporal Association Cortex Right | 66 | 492 | 13% | 7.27 |
| Hippocampal Formation CA2 Field Right | 152 | 317 | 48% | 7.27 |
| Perirhinal Cortex Left | 148 | 730 | 20% | 7.22 |
| Auditory Cortex Secondary Left | 93 | 397 | 23% | 7.22 |
| Perirhinal Cortex Right | 116 | 759 | 15% | 7.21 |
| Thalamus Ventral Medial Nucleus Left | 48 | 109 | 44% | 7.12 |
| Thalamus Posterior Nucleus Left | 115 | 337 | 34% | 7.09 |
| Extended Amygdala Medial Division Left | 44 | 490 | 9% | 7.08 |
| Somatosensory Cortex Primary Left | 68 | 381 | 18% | 7.08 |
| Stria Terminalis Left | 58 | 89 | 65% | 7.07 |
| Auditory Cortex Secondary Right | 116 | 413 | 28% | 7.03 |
| Thalamus Intralaminar Nuclei Left | 77 | 243 | 32% | 7.03 |
| Thalamus Intralaminar Nuclei Right | 50 | 236 | 21% | 7.01 |
| Pretectum Left | 64 | 239 | 27% | 7.00 |
| Subthalamic Nucleus Right | 22 | 22 | 100% | 6.99 |
| Reticular Thalamic Nucleus Left | 53 | 269 | 20% | 6.94 |
| Corpus Callosum Left | 79 | 1892 | 4% | 6.93 |
| Auditory Cortex Primary Right | 232 | 623 | 37% | 6.87 |
| Substantia Nigra Left | 84 | 195 | 43% | 6.85 |
| Cerebellum Lobule 04 & Lobule 05 Left | 34 | 344 | 10% | 6.84 |
| Thalamus Lateral Nucleus Right | 94 | 330 | 28% | 6.84 |
| Somatosensory Cortex Secondary Right | 61 | 887 | 7% | 6.84 |
| Inferior Colliculus Left | 32 | 800 | 4% | 6.83 |
| Pretectum Right | 150 | 228 | 66% | 6.82 |
| Superior Colliculus Right | 72 | 855 | 8% | 6.78 |
| Optic Nuclei Accessory Left | 12 | 12 | 100% | 6.75 |
| Striatum Dorsal Left | 88 | 2939 | 3% | 6.73 |
| Auditory Cortex Primary Left | 168 | 601 | 28% | 6.71 |
| Olfactory Cortex Lateral Right | 48 | 3351 | 1% | 6.66 |
| Reticular Formation Midbrian Right | 60 | 547 | 11% | 6.63 |
| Thalamus Ventral Medial Nucleus Right | 33 | 105 | 31% | 6.54 |
| Extended Amygdala Medial Division Right | 38 | 491 | 8% | 6.53 |
| Hippocampal Formation Dentate Gyrus Left | 47 | 890 | 5% | 6.52 |
| Thalamus Lateral Nucleus Left | 33 | 332 | 10% | 6.46 |
