# Supplementary material for: Robust Reproducible Resting State Networks in the Awake Rodent Brain
Source: PLoS One. 2011 Oct 18;6(10):e25701. doi: 10.1371/journal.pone.0025701 (PMC3196498; doi:10.1371/journal.pone.0025701)
Supplement: Table S6 — Table of Activations for Component 6. The Table lists the most significant activated structures for the Sensory Network. Structures were identified using the Paxinos Atlas [33]. Structures are listed according to the fraction of the structure being active and the statistical significance of the activation (See Methods Section). (DOCX) [file pone.0025701.s009.docx]

**Table 6: Component 6 - Sensory (Exteroceptive) Network**

| **Brain Structure** | **Active** | **Total** | **%Active** | **Avg Z** |
| --- | --- | --- | --- | --- |
| Somatosensory Cortex Primary Jaw Region Oral Surface Left | 159 | 162 | 98% | 9.45 |
| Somatosensory Cortex Primary Jaw Region Left | 567 | 629 | 90% | 8.83 |
| Somatosensory Cortex Primary Upper Lip Region Left | 441 | 466 | 95% | 8.25 |
| Somatosensory Cortex Primary Jaw Region Oral Surface Right | 158 | 159 | 99% | 7.86 |
| Motor Cortex Primary Left | 1152 | 1830 | 63% | 7.80 |
| Somatosensory Cortex Primary Upper Lip Region Right | 429 | 467 | 92% | 7.71 |
| Somatosensory Cortex Primary Jaw Region Right | 563 | 688 | 82% | 7.62 |
| Somatosensory Cortex Secondary Left | 808 | 918 | 88% | 7.59 |
| Motor Cortex Secondary Left | 683 | 1278 | 53% | 7.45 |
| Motor Cortex Primary Right | 782 | 1643 | 48% | 7.36 |
| Somatosensory Cortex Primary Dysgranular Region Left | 163 | 232 | 70% | 7.31 |
| Somatosensory Cortex Primary Left | 300 | 381 | 79% | 7.23 |
| Somatosensory Cortex Secondary Right | 667 | 887 | 75% | 7.22 |
| Somatosensory Cortex Primary Right | 147 | 385 | 38% | 7.20 |
| Somatosensory Cortex Primary Barrel Field Left | 1135 | 1590 | 71% | 7.14 |
| Somatosensory Cortex Primary Dysgranular Region Right | 156 | 233 | 67% | 6.97 |
| Insular Cortex Left | 618 | 1259 | 49% | 6.95 |
| Motor Cortex Secondary Right | 601 | 1249 | 48% | 6.92 |
| Insular Cortex Right | 302 | 1228 | 25% | 6.86 |
| Somatosensory Cortex Primary Forelimb Region Right | 412 | 632 | 65% | 6.81 |
| Auditory Cortex Secondary Left | 107 | 397 | 27% | 6.80 |
| Somatosensory Cortex Primary Barrel Field Right | 925 | 1600 | 58% | 6.70 |
| Orbitofrontal Cortex Left | 119 | 1142 | 10% | 6.67 |
| Somatosensory Cortex Primary Forelimb Region Left | 404 | 644 | 63% | 6.63 |
| Auditory Cortex Secondary Right | 35 | 413 | 8% | 6.62 |
| Orbitofrontal Cortex Right | 228 | 1092 | 21% | 6.50 |
| Cingulate Cortex Left | 92 | 953 | 10% | 6.47 |
| Corpus Callosum Left | 349 | 1892 | 18% | 6.46 |
| Corpus Callosum Right | 196 | 1863 | 11% | 6.41 |
| Temporal Association Cortex Left | 64 | 462 | 14% | 6.29 |
| Perirhinal Cortex Left | 63 | 730 | 9% | 6.21 |
| Striatum Dorsal Left | 109 | 2939 | 4% | 6.14 |
| Perirhinal Cortex Right | 48 | 759 | 6% | 6.11 |
| Fimbria Fronix Left | 61 | 604 | 10% | 6.10 |
| Striatum Dorsal Right | 75 | 2932 | 3% | 6.07 |
| Cingulate Cortex Right | 52 | 994 | 5% | 6.01 |
| Hippocampal Formation CA3 Field Left | 80 | 725 | 11% | 5.96 |
| Olfactory Cortex Lateral Right | 147 | 3351 | 4% | 5.92 |
| Auditory Cortex Primary Left | 54 | 601 | 9% | 5.92 |
| Olfactory Nucleus Anterior Right | 63 | 894 | 7% | 5.90 |
| Somatosensory Cortex Primary Hindlimb Region Left | 127 | 304 | 42% | 5.89 |
| Olfactory Nucleus Anterior Left | 56 | 874 | 6% | 5.89 |
| Extended Amygdala Central Division Left | 34 | 471 | 7% | 5.85 |
| Olfactory Cortex Lateral Left | 38 | 3380 | 1% | 5.83 |
