# Supplementary material for: Robust Reproducible Resting State Networks in the Awake Rodent Brain
Source: PLoS One. 2011 Oct 18;6(10):e25701. doi: 10.1371/journal.pone.0025701 (PMC3196498; doi:10.1371/journal.pone.0025701)
Supplement: Table S7 — Table of Activation for Component 7. The Table lists the most significant activated structures for the Interoceptive Network. Structures were identified using the Paxinos Atlas [33]. Structures are listed according to the fraction of the structure being active and the statistical significance of the activation (See Methods Section). (DOCX) [file pone.0025701.s010.docx]

**Table 7: Component 7 - Interoceptive Network**

| **Brain Structure** | **Active** | **Total** | **% Active** | **Avg Z** |
| --- | --- | --- | --- | --- |
| Somatosensory Cortex Primary Trunk Region Right | 464 | 473 | 98% | 8.22 |
| Somatosensory Cortex Primary Dysgranular Region Right | 199 | 233 | 85% | 7.89 |
| Somatosensory Cortex Primary Hindlimb Region Left | 245 | 304 | 81% | 7.89 |
| Somatosensory Cortex Primary Forelimb Region Right | 544 | 632 | 86% | 7.78 |
| Somatosensory Cortex Primary Forelimb Region Left | 480 | 644 | 75% | 7.67 |
| Somatosensory Cortex Primary Dysgranular Region Left | 195 | 232 | 84% | 7.52 |
| Somatosensory Cortex Primary Hindlimb Region Right | 250 | 309 | 81% | 7.51 |
| Somatosensory Cortex Primary Trunk Region Left | 390 | 458 | 85% | 7.43 |
| Parietal Cortex Posterior Area Right | 248 | 281 | 88% | 7.31 |
| Somatosensory Cortex Primary Barrel Field Right | 1354 | 1600 | 85% | 7.23 |
| Motor Cortex Primary Right | 707 | 1643 | 43% | 7.06 |
| Motor Cortex Secondary Right | 384 | 1249 | 31% | 7.02 |
| Insular Cortex Right | 68 | 1228 | 6% | 6.98 |
| Somatosensory Cortex Primary Barrel Field Left | 1060 | 1590 | 67% | 6.91 |
| Insular Cortex Left | 73 | 1259 | 6% | 6.90 |
| Motor Cortex Primary Left | 607 | 1830 | 33% | 6.90 |
| Somatosensory Cortex Primary Jaw Region Right | 187 | 688 | 27% | 6.82 |
| Somatosensory Cortex Secondary Right | 346 | 887 | 39% | 6.79 |
| Retrosplenial Cortex Right | 441 | 1134 | 39% | 6.78 |
| Somatosensory Cortex Primary Right | 290 | 385 | 75% | 6.78 |
| Auditory Cortex Secondary Right | 78 | 413 | 19% | 6.72 |
| Motor Cortex Secondary Left | 352 | 1278 | 28% | 6.67 |
| Cingulum Right | 83 | 154 | 54% | 6.64 |
| Parietal Cortex Posterior Area Left | 126 | 290 | 43% | 6.60 |
| Somatosensory Cortex Primary Jaw Region Left | 87 | 629 | 14% | 6.59 |
| Visual Cortex Secondary Right | 324 | 1312 | 25% | 6.59 |
| Auditory Cortex Primary Right | 142 | 623 | 23% | 6.56 |
| Somatosensory Cortex Secondary Left | 419 | 918 | 46% | 6.49 |
| Somatosensory Cortex Primary Upper Lip Region Right | 291 | 467 | 62% | 6.49 |
| Cingulate Cortex Right | 255 | 994 | 26% | 6.48 |
| Retrosplenial Cortex Left | 243 | 934 | 26% | 6.39 |
| Somatosensory Cortex Primary Jaw Region Oral Surface Left | 93 | 162 | 57% | 6.35 |
| Perirhinal Cortex Left | 33 | 730 | 5% | 6.34 |
| Corpus Callosum Right | 302 | 1863 | 16% | 6.28 |
| Fimbria Fronix Right | 52 | 550 | 9% | 6.26 |
| Cingulum Left | 68 | 155 | 44% | 6.23 |
| Cingulate Cortex Left | 204 | 953 | 21% | 6.23 |
| Somatosensory Cortex Primary Jaw Region Oral Surface Right | 50 | 159 | 31% | 6.17 |
| Olfactory Cortex Lateral Left | 111 | 3380 | 3% | 6.17 |
| Somatosensory Cortex Primary Left | 112 | 381 | 29% | 6.16 |
| Cerebellum Lobule 04 & Lobule 05 Left | 38 | 344 | 11% | 6.10 |
| Hippocampal Formation Dentate Gyrus Left | 79 | 890 | 9% | 6.10 |
| Hippocampal Formation CA1 Field Right | 65 | 973 | 7% | 6.08 |
| Orbitofrontal Cortex Right | 95 | 1092 | 9% | 6.06 |
| Corpus Callosum Left | 80 | 1892 | 4% | 6.05 |
| Somatosensory Cortex Primary Upper Lip Region Left | 83 | 466 | 18% | 5.99 |
| Visual Cortex Secondary Left | 47 | 1244 | 4% | 5.95 |
| Auditory Cortex Primary Left | 43 | 601 | 7% | 5.80 |
| Inferior Colliculus Left | 34 | 800 | 4% | 5.77 |
| Striatum Dorsal Right | 38 | 2932 | 1% | 5.73 |
